# BeStSel: analysis site for protein CD spectra—2025 update

**DOI:** 10.1093/nar/gkaf378

**Published:** 2025-05-13

**Authors:** András Micsonai, Frank Wien, Nikoletta Murvai, Márton Péter Nyiri, Bori Balatoni, Young-Ho Lee, Tamás Molnár, Yuji Goto, Frédéric Jamme, József Kardos

**Affiliations:** Department of Biochemistry, Institute of Biology, ELTE Eötvös Loránd University, Budapest H-1117, Hungary; ELTE—Functional Nucleic Acid Motifs Research Group, Department of Biochemistry, Institute of Biology, ELTE Eötvös Loránd University, Budapest H-1117, Hungary; Synchrotron SOLEIL, Gif-sur-Yvette 91192, France; Department of Biochemistry, Institute of Biology, ELTE Eötvös Loránd University, Budapest H-1117, Hungary; ELTE—Functional Nucleic Acid Motifs Research Group, Department of Biochemistry, Institute of Biology, ELTE Eötvös Loránd University, Budapest H-1117, Hungary; Department of Biochemistry, Institute of Biology, ELTE Eötvös Loránd University, Budapest H-1117, Hungary; Department of Biochemistry, Institute of Biology, ELTE Eötvös Loránd University, Budapest H-1117, Hungary; Biopharmaceutical Research Center, Korea Basic Science Institute (KBSI), Cheongju 28119, Republic of Korea; Bio-Analytical Science, University of Science and Technology, Daejeon 34113, Republic of Korea; Graduate School of Analytical Science and Technology, Chungnam National University, Daejeon 34134, Republic of Korea; Department of Systems Biotechnology, Chung-Ang University, Gyeonggi 17546, Republic of Korea; Frontier Research Institute for Interdisciplinary Sciences, Tohoku University, Sendai 980-8578, Japan; Department of Biochemistry, Institute of Biology, ELTE Eötvös Loránd University, Budapest H-1117, Hungary; ELTE NAP Neuroimmunology Research Group, Department of Biochemistry, Institute of Biology, ELTE Eötvös Loránd University, Budapest H-1117, Hungary; Graduate School of Engineering, Osaka University, Osaka 565-0871, Japan; Synchrotron SOLEIL, Gif-sur-Yvette 91192, France; Department of Biochemistry, Institute of Biology, ELTE Eötvös Loránd University, Budapest H-1117, Hungary; ELTE NAP Neuroimmunology Research Group, Department of Biochemistry, Institute of Biology, ELTE Eötvös Loránd University, Budapest H-1117, Hungary

## Abstract

Circular dichroism (CD) spectroscopy is a widely used technique to characterize the secondary structure composition of proteins. We have developed the Beta Structure Selection (BeStSel) method (PNAS, 112, E3095), which solves the main problem of protein CD spectroscopy—namely, the spectral variability of β-structures. The BeStSel web server utilizes this method to provide tools to the community for CD spectrum analysis. BeStSel uniquely provides information on eight secondary structure components, including parallel β-structure and antiparallel β-sheets with three different twist groups. It outperforms all available methods in accuracy and information content, and is also able to predict protein folds down to the topology/homology level of the CATH classification. The algorithm has been further developed, and the accuracy of the estimation of the secondary structure elements is improved by 0.7% as an average on the reference dataset. A new module of the web server calculates protein stability from the thermal denaturation profile followed by CD. Secondary structure calculations of uploaded PDB and mmcif files support the experimental verification of MD simulations and AlphaFold models by CD spectroscopy. Well-proven modules for disorder–order classification and extinction coefficient calculation continue to work. The BeStSel server is freely accessible at https://bestsel.elte.hu.

## Graphical Abstract

**Graphical Abstract:**
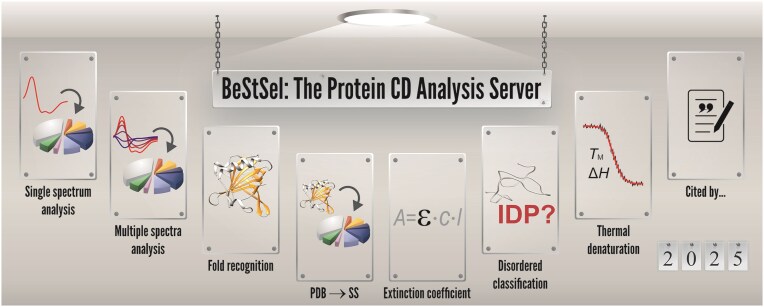

## Introduction

Circular dichroism (CD) spectroscopy in the far-UV region is commonly used to investigate the secondary structure of proteins. It is a valuable tool when a rapid and cost-effective technique is needed, or when the application of high-resolution methods [such as X-ray crystallography or nuclear magnetic resonance (NMR)] is impractical. CD spectroscopy has broad applications across various fields of protein science. It can be used to verify the correct folding of recombinant proteins, assess the impact of environmental factors (such as pH, ionic strength, additives, and crowding), and study how protein modifications (e.g. mutations and post-translational modifications) influence structure and stability. Furthermore, CD spectroscopy serves as an effective experimental approach to validate structural predictions made by bioinformatics tools that rely on the ever-expanding protein databases.

While the instrumentation is well established and routinely used, the major challenge in protein CD spectroscopy is understanding the spectral contributions of different secondary structural elements. Over the past few decades, numerous algorithms have been created to extract secondary structure information from CD spectra. However, accurate structural estimation has mostly been limited to α-helical proteins due to the considerable spectral variability exhibited by β-sheet-containing proteins [1, 2]. We have demonstrated that the parallel–antiparallel arrangement and the twist of β-sheets account for this spectral diversity and have developed the Beta Structure Selection (BeStSel) method for precise secondary structure determination. BeStSel provides detailed structural information, distinguishing eight structural components, and outperforms all other methods in terms of accuracy [3]. Additionally, it has the unique capability to predict protein folds at the topology and homology level of the CATH protein fold classification [4]. The BeStSel web server, freely accessible to the scientific community, enables rapid, user-friendly, and accurate analysis of CD spectra. In this paper, we present the latest developments and the current status of the BeStSel web server.

## Materials and methods (web server description)

### Secondary structure components and the twist of β-sheets

BeStSel defines eight secondary structure components based on the Dictionary of Secondary Structure of Proteins (DSSP) classification [5]. Residues assigned to α-helix by DSSP are divided into regular (Helix1) and distorted (Helix2) categories, as the middle part and the two–two residues at both ends of the helices, respectively. β-Strand residues are classified into four β-sheet types: (i) parallel β-sheet and antiparallel β-sheets with varying degrees of twist—(ii) left-hand twisted (Anti1), (iii) relaxed, slightly right-hand twisted (Anti2), and (iv) right-hand twisted (Anti3) [3]. Turn regions follow the DSSP definition, while all remaining residues, including unassigned ones, are grouped under “Others.”

### New “basis matrices” and fitting to the CD spectrum

Although the combination of the original basis spectra of the eight components of BeStSel [3] describe the overall CD spectrum better than other algorithms do [3,6], eight components are still a simplification and the basis spectra sets represent average basis spectra optimized for the entire reference dataset of proteins with largely different secondary structure compositions and spectral components that differ from the average basis spectra. For example, the spectral shape of β-sheets show a continuous distribution depending on the twist; four groups cannot describe the contribution of β-sheets perfectly [3]. Turn is an optimized “average” turn; however, its spectrum might also vary with the protein. The “others” component collects several different secondary structure elements, with contributions specific for the individual protein. Moreover, probably at a lesser extent, we cannot exclude the effect of secondary structure elements on each other in the tertiary structure of the protein. Thus, as an example, the optimal basis spectra will be different for a protein with high α-helix content or with a specific β-sheet composition. Based on these considerations, the basis spectra sets could be further optimized to be more specific for the actual secondary structure composition of the protein, i.e. its localization in the secondary structural space. To address this issue, instead of fixed basis spectra, we introduced “basis matrices,” which handle spectral contributions depending on the location in the secondary structure space. The CD signal at a given wavelength can be expressed as follows:


(1) \begin{eqnarray*}
C{D_\lambda } = S{S^{\prime}}\, \cdot \,{M_\lambda } \cdot SS
\end{eqnarray*}

Where *SS* and *SS*′ are the secondary structure vector and its transpose containing the fractions for the eight secondary BeStSel structure elements of the protein, and ***M***_λ_ is the basis matrix at λ wavelength.

To optimize ***M***_λ_, the following two least-squares problem has to be solved for all the eight secondary structure (*i = 1,...,8*), on subsets of the reference dataset of proteins with high quality CD spectra and known atomic resolution structure.


(2) \begin{eqnarray*}
\min \left( {\frac{1}{2}{{\left\| {SS_{ref}^{\prime} \cdot {b_\lambda } - C{D_{ref,\lambda }}} \right\|}^2}} \right),
\end{eqnarray*}

(3) \begin{eqnarray*}
\min \left( {\sum\nolimits_{j = 1}^n {{{\left( {SS_j^{\prime} \cdot M{{\left( {:,i} \right)}_\lambda } - {b_{i,\lambda }}} \right)}^2}} } \right),
\end{eqnarray*}

where *SS*_ref_ and *CD*_ref_ are an *n*-by-8 matrix of secondary structure contents and a column vector of CD signals at the corresponding λ wavelength of *n* reference proteins, respectively. ***b*_λ_** is a vector containing the calculated CD values for eight basis spectra at λ wavelength, that the linear least-squares problem, Eq. (2) is solved for. ***b***_i,λ_ is the optimized CD value of basis spectra of *i*th secondary structure at λ wavelength (from Eq. 2), *SS*_j_ is a vector of secondary structure content of *j*th reference protein, and ***M***(:,*i*)_λ_ is the *i*th column of basis matrix ***M***_λ_, containing the coefficients the linear least-squares problem, Eq. (3) is solved for. $\| x \|$ is the Euclidean norm for vector *x*.

For optimizing basis matrix ***M*** for any secondary structure, a separate subset of the reference dataset was generated for every secondary structure component *i*, and every wavelength λ by subsequently leaving out proteins one by one, in the absence of which the secondary structure prediction using the calculated basis matrix ***M*** and the conditional least-squares problem Eq. (4) on the entire reference set improved most significantly for that particular secondary structure component.


(4) \begin{eqnarray*}
&&\min \left( {\frac{1}{2}{{\left\| {S{S_{cal{c^{\prime}}}}\, \cdot M \cdot S{S_{calc}} - C{D_{exp}}} \right\|}^2}} \right),\nonumber\\ &&{\mathrm{where }}\sum\nolimits_{i = 1}^N {S{S_{calc,i}} = 1} \,\,{\mathrm{and}}\,\,S{S_{calc,i}} \ge 0,\,i = 1\, \ldots N
\end{eqnarray*}

To fit to the measured CD signal of an unknown protein, the same Eq. (4) must be solved for *SS_calc_* using the predetermined ***M***.

### Protein fold prediction by BeStSel

Protein folds can be characterized by distinct secondary structure patterns. The eight secondary structure components defined by BeStSel provide sufficient structural information to predict protein fold. The β-sheet composition, including the ratio of parallel and antiparallel β-sheets and the degree of twist in antiparallel β-sheets, effectively differentiates β-structure-containing folds, while the two α-helix components reflect the number and average length of α-helices [3]. BeStSel applies the CATH protein fold classification system [7], which organizes protein structures hierarchically into class, architecture, topology, and homology (superfamily) levels. CATH’s advantage lies in its extensive, continuously updated coverage of protein domains in the PDB. Every protein structure in the PDB can be represented by BeStSel’s secondary structure components as a point in an eight-dimensional secondary structure space, and so do the results of the BeStSel CD spectrum analysis. Fold prediction involves locating PDB structures with secondary structure compositions similar to the results of the CD spectrum analysis, based on their Euclidean distance in this eight-dimensional space, and determining their CATH fold classification. However, the regions that protein folds occupy in the fold space might overlap making accurate identification challenging.

BeStSel offers four-fold prediction methods:

- Search for closest neighbors: Identifies the 20 closest structures in Euclidean space on the entire PDB and lists their CATH classifications. In this search, hits of multidomain proteins with domains of various folds can show the same average secondary structure contents and make the prediction difficult.
  For single domain proteins, the fold prediction is more feasible. A reference dataset containing 73 535 nonredundant (≤95% sequence identity) single-domain structures from CATH 4.4 [4] is used. This subset spans five-fold classes, 43 architectures, 1472 topologies, and 6630 homologies, with their secondary structure compositions derived from corresponding PDB structures. The following prediction methods can be applied:
- Search for the closest structures: The ten closest structures based on the Euclidean distance in the eight dimensional space of BeStSel is listed for their CATH classifications, useful for rare fold types.
- Search within the expected error of CD analysis: Finds all structures within 1.5 × RMSD of BeStSel’s average error in each secondary structure components based on the performance on the reference dataset. The search lists the hits by their frequency for different folds at the four CATH levels. This method is particularly usable in densely populated fold regions where hundreds of hits may be identified and the closest hits are not necessarily the correct ones.
- Weighted *k*-nearest neighbors (WKNN): Predicts the class, architecture, topology, and homology of the query secondary structure composition. In each category, potential folds are ranked by WKNN score [8, 9].

### Disordered-ordered binary classification

A total of 262 CD spectra of ordered and disordered proteins were obtained from the Protein Circular Dichroism Data Bank (PCDDB) [10], generated from in-house measurements [11], or sourced from the literature [12]. The classification employs a *k*-nearest neighbor (k-NN) model with a cosine distance function [13], using CD data at of three wavelengths, either 197–206–233 nm or 212–217–225 nm. The distinction between ordered and disordered proteins is determined based on the analysis of the 10 nearest neighbors in the reference dataset [12].

### Calculation of protein stability from thermal denaturation profile

CD spectroscopy is a perfect technique to follow the conformational changes of proteins and thus follow protein unfolding and testing the conformational stability of proteins. The CD spectrum is characteristic to the secondary structure composition and is not sensitive to the environmental factors, such as temperature (provided that the protein structure does not change). This makes CD an excellent technique to follow the thermal stability of proteins versus fluorescence techniques where the fluorescence efficiency itself is highly temperature dependent. Thermal unfolding profile can be followed by collecting series of spectra or by following the CD signal at a carefully chosen constant wavelength as a function of temperature. Now, the web server supports the analysis of thermal denaturation profiles recorded at a constant wavelength by fitting a two-state model to it, described by Shih *et al.* [14]. The calculation is based on the Gibbs–Helmholtz equation and provides the *T*_m_ melting temperature and the Δ*H*_m_ enthalpy change for unfolding at the melting temperature by fitting with the following function [14]:


(5) \begin{eqnarray*}
C{D_{exp}}\left( T \right) = \frac{{{A_N} + {m_N} \cdot T + \left[ {\left( {{A_D} + {m_D} \cdot T} \right) \cdot \exp \left( y \right) \cdot {{\left( {\frac{T}{{{T_m}}}} \right)}^{\Delta Cp/R}}} \right]}}{{1 + \exp \left( y \right) \cdot {{\left( {\frac{T}{{{T_m}}}} \right)}^{\Delta Cp/R}}}}
\end{eqnarray*}

where:


(6) \begin{eqnarray*}
y = \frac{1}{R} \cdot \left( {\frac{{{T_m} \cdot \Delta {C_p} - \Delta {H_m}}}{T} + \frac{{\Delta {H_m}}}{{{T_m}}} - \Delta {C_p}} \right)
\end{eqnarray*}

*A_N_*, *A_D_*, *m_N_*, and *m_D_* are the starting amplitudes of the native and denatured states and their temperature dependence, i.e. the slopes of the denaturation curve before and after the melting transition, respectively. *T* is the absolute temperature (K) and *R* is the universal gas constant. Δ*C*_p_ is not fitted at the web server its value can be set in the fitting function. Having an experimentally determined or estimated Δ*C*_p_, the heat capacity difference between the denatured and native state, one can calculate the free energy change of unfolding (Δ*G*_N-U_) that describes protein stability (which is its negative), at any temperature values:


(7) \begin{eqnarray*}
\Delta G\left( T \right) = \Delta {H_m} \cdot \left( {1 - \frac{T}{{{T_m}}}} \right) - \Delta {C_P} \cdot \left[ {\left( {{T_m} - T} \right) + T \cdot ln\left( {\frac{T}{{{T_m}}}} \right)} \right]
\end{eqnarray*}

For globular proteins, Δ*C*_p_ is associated with, and can be estimated from the change in the accessible polar and apolar surface areas and in the total buried surface area upon unfolding [15] (for small globular proteins, Δ*C*_p_ is ∼50 J/mol/K per residue).

### The operation of the BeStSel web server

Under the *Documentation* tab, information on the web server is presented. A tutorial in pdf providing a detailed guide can be downloaded. A brief Guide to CD measurements and data processing, and FAQ is also available.

Using the web server, error messages notify when input data format is not suitable. Warning messages appear in case of potential issues, such as abnormal spectral amplitudes, which can be a result of improper CD unit choice or data normalization.

The server provides eight program modules: *Single spectrum analysis*, *Multiple spectra analysis*, *Fold recognition*, *Secondary structure from PDB files*, *Extinction coefficient calculation* from the primary sequence, *Disordered-ordered binary classification*, *Thermal denaturation analysis*, and a searchable collection of publications using CD spectroscopy with BeStSel analysis.

In *Single-spectrum analysis*, users can upload and analyze a CD spectrum using the BeStSel method to determine secondary structure contents. Data can be pasted as two columns in a text window or uploaded as a .txt file. The program automatically detects file headers and accepts data with pitches of 0.1, 0.2, 0.5, or 1 nm. Data are smoothed as data average using a 2 nm window and converted to data with 1 nm intervals (see explanation on new NRMSD later). Measurement files from various instruments, saved in text format, are properly processed by the server. Accepted input units include Δϵ (M^-1^cm^-1^), [Θ] (mean residue ellipticity in deg cm^-2^ dmol^-1^), or measured ellipticity (mdeg). For the latter, users must provide concentration, residue number, and pathlength for normalization. The uploaded data will be transformed to Δϵ units.

After clicking on the submit button, a “Data Examination” window appears to confirm successful data upload. With a subsequent click, the secondary structure contents are calculated based on BeStSel’s eight secondary structure components, and the results are displayed graphically alongside spectral fitting metrics, including root mean square deviation and normalized RMSD (NRMSD). The initial fitting is performed over the broadest wavelength range, after which users can adjust the lower wavelength limit in 5 nm increments and recalculate the secondary structure content. The output image can be customized, redrawn, or saved as a .txt or .csv file for further analysis or figure preparation.

The software also allows spectrum rescaling with a user-defined factor, and the “Best factor” function performs multiple recalculations using re-scaling between 0.5- and 2-folds the value set as scale factor. This feature helps assess the influence of CD amplitude on fitting accuracy and may reveal errors in concentration determination or data normalization. However, the factor yielding the lowest NRMSD should not be considered a correction for normalized spectra when analyzed in the 190–250 nm or 200–250 nm ranges. Accurate concentration input remains essential for reliable secondary structure analysis.

The *Multiple Spectra Analysis* function enables the simultaneous analysis of a series of CD spectra, which is particularly useful for datasets collected under varying conditions, such as ligand or denaturant concentration, temperature, time, etc. Data can be pasted into the text window or uploaded from a text file, with input units identical to those in Single Spectrum Analysis. Following “Data Examination,” secondary structure contents are computed with a single click and presented as graphical output or saved as .txt or .csv files. The wavelength range and scaling factor can be adjusted for recalculations, similar to the options in *Single Spectrum Analysis*.

*Fold recognition* based on secondary structure content can be initiated with a single click, performing four types of analysis as outlined in the “Materials and methods” section. The fold recognition module can also be used independently of CD spectrum analysis by manually inputting the eight secondary structure components and chain length. The output includes the top-ranked 1, 5, 10, and 15 CATH classes, architectures, topologies, and homologies. For structural studies, including the analysis of model structures or PDB-derived structures, the WKNN method is recommended for optimal fold identification.

In *Thermal denaturation analysis*, thermal denaturation profiles in the form of .txt files containing columns of temperature and CD values can be uploaded, or data columns can be copied into the window and submit with a single click. The server automatically analyses the uploaded data, provides the results graphically and show the fitting parameters. The temperature range can be selected and the fitting recalculated. Fitting parameters can be manually adjusted for recalculation, as well. Adjusting the value of Δ*C*_p_, the protein stability is calculated and shown for 25°C and 37°C.

The “*Secondary Structure from PDB Files*” module calculates the eight BeStSel components and provides, for comparison, the secondary structure composition based on DSSP [5] and SELCON3 [16]. For structures in the Protein Data Bank (PDB), users can input the four-letter PDB ID, and the program will also retrieve corresponding CATH classification if available. Alternatively, structural files in PDB or mmCIF format (up to 20 MB) can be uploaded, and the calculation will proceed automatically. Results are available in both graphical and text formats. This module is particularly valuable for validating molecular dynamics (MD) simulations or AlphaFold models [17] through comparison with experimental CD spectroscopy data (see “Results and discussion” section).

The *Extinction Coefficient Calculation* module computes protein and peptide extinction coefficients at 214 nm [18] and 205 nm [19], based on the primary sequence and disulfide bond count. Users can input or paste the amino acid sequence into the text window. The resulting extinction coefficients facilitate direct concentration determination of the protein sample upon the CD measurement.

The *Disordered-Ordered Classification* module analyzes far-UV CD data to detect disordered structures based on CD values at three specific wavelengths (197–206–233 nm or 212–217–225 nm). Users can paste the data into the text window, with the first column for wavelength and subsequent columns for spectral data; full spectra, multiple spectra, or data limited to the required wavelengths are accepted. The output table contains the wavelength triplets used for analysis and the predicted classification.

The *Cited by…* feature opens a separate window displaying a database of scientific articles that have employed CD spectroscopy with BeStSel analysis. The database includes article identifiers and keywords, allowing users to search and explore practical examples of CD spectroscopy applications with BeStSel.

## Results and discussion

### Performance

While CD spectroscopy instrumentation is well advanced, extracting structural information from CD spectra remains a significant challenge. The development of the BeStSel method addresses this issue by resolving the structural diversity of β-structures. BeStSel’s eight structural components offer more accurate secondary structure estimation than previous methods [3]. Notably, the method enables reliable structural analysis of β-sheet-rich proteins, including antibodies, membrane proteins, aggregates, and amyloid fibrils. Additionally, BeStSel provides sufficient structural information to predict protein folds down to the topology/homology levels of the CATH classification.

The most important present upgrade is the introduction of the new basis spectrum system, optimization method and the new procedure to fitting the CD spectra.

Table 1 compares the updated BeStSel method with its previous version on the reference database, demonstrating improved accuracy. For instance, in the 175–250 nm range, the RMSD for α-helix, overall antiparallel-β and “others” estimation decreased from 0.041 to 0.036, from 0.064 to 0.041, and from 0.052 to 0.042, respectively. Performance was also evaluated on an independent set of β-sheet-rich and rare protein structures and compared to other secondary structure estimation methods (Supplementary Table S1). When recalculated to common structural categories (helix, antiparallel β, parallel β, overall β-sheet, and “turn + others”), BeStSel achieved RMSDs of 0.035, 0.046, 0.031, 0.034, and 0.038, respectively, outperforming other methods, which showed RMSDs ranging from 0.083 to 0.26, 0.12 to 0.214, 0.076 to 0.198, 0.068 to 0.23, and 0.074 to 0.232.

**Table 1.** Secondary structure estimation performance of the updated BeStSel web server^a^

	175–250 nm				190–250 nm				200–250 nm			
	BeStSel 2022		BeStSel 2025		BeStSel 2022		BeStSel 2025		BeStSel 2022		BeStSel 2025	
	RMSD	Corr	RMSD	Corr	RMSD	Corr	RMSD	Corr	RMSD	Corr	RMSD	Corr
Helix1	0.027	0.98	0.023	0.99	0.028	0.98	0.025	0.98	0.030	0.98	0.026	0.99
Helix2	0.025	0.93	0.019	0.96	0.026	0.92	0.027	0.92	0.029	0.90	0.028	0.90
Anti1	0.016	0.87	0.013	0.93	0.018	0.83	0.012	0.94	0.026	0.62	0.014	0.48
Anti2	0.034	0.92	0.024	0.96	0.035	0.92	0.033	0.94	0.035	0.92	0.025	0.96
Anti3	0.041	0.88	0.027	0.95	0.040	0.88	0.034	0.92	0.046	0.85	0.034	0.92
Parallel	0.041	0.91	0.031	0.95	0.041	0.91	0.031	0.95	0.039	0.91	0.034	0.94
Turn	0.033	0.74	0.025	0.83	0.032	0.73	0.026	0.85	0.032	0.72	0.030	0.76
Others	0.052	0.83	0.042	0.90	0.058	0.78	0.046	0.88	0.063	0.74	0.047	0.87
Helix	0.041	0.98	0.036	0.99	0.042	0.98	0.044	0.98	0.045	0.98	0.044	0.98
Antiparallel	0.064	0.94	0.041	0.97	0.064	0.94	0.054	0.96	0.072	0.92	0.065	0.93
Beta	0.056	0.94	0.040	0.97	0.057	0.94	0.056	0.95	0.065	0.92	0.058	0.94
Turn + Others	0.053	0.88	0.040	0.93	0.056	0.84	0.047	0.90	0.061	0.82	0.045	0.90

^a^Cross validated statistics evaluated on the SP175 + reference database [3,10]. Root-mean-square-deviation and Pearson correlation for different wavelength ranges are provided for the previous [6] and the updated BeStSel methods for three wavelength ranges.

The functionality of the BeStSel web server was compared to other online tools in our previous work [6]. After 10 years of run, BeStSel has become well-known and because of its accuracy and user-friendly interface, now BeStSel is the most popular tool for CD spectroscopy users proven by the number of citations.

### Applications

CD spectroscopy combined with BeStSel analysis is widely applied across various fields of protein science. Since its introduction in 2015 [3], BeStSel has been utilized in over 2000 scientific studies. A searchable database of these publications is available on the web server, offering practical examples for users. Here, we present some recent, notable examples.

Although CD spectroscopy is a low-resolution technique, combined with BeStSel analysis it is frequently used as a complementary method to high-resolution techniques. Maus *et al.* investigated ZIKV inhibitors using NMR, with CD and BeStSel serving as complementary approaches [20]. Similar methodologies have been employed by Cunha *et al.* on human serum albumin—nonsteroidal anti-inflammatory drug interaction where CD indicated a decrease in α-helix content [21], and Parron-Ballesteros *et al.* investigating the molecular background of allergenic effect of Sola l 7, a class I lipid transfer protein found in tomato seeds that has been identified as an allergen linked to severe anaphylaxis [22].

BeStSel has been used in protein expression, purification and quality control studies. For example, Geens *et al.* characterized the N-terminal domain of the Plasmodium falciparum circumsporozoite protein [23]. Kinkar *et al.* analyzed the structure of mosquito larvae proteins using BeStSel [24].

CD is widely used to assess protein stability. Bozkurt *et al.* investigated the glycosylation of alkaline phosphatase, analyzing the protein’s thermal stability using CD combined with BeStSel [25]. Schwidetzky investigated the properties of fungal ice-nucleator proteins [26]. Heat stability of thermophilic ATP synthase’s β subunit was studied by López-Pérez *et al.* [27].

The effect of additives and buffer conditions on the structure of different proteins was studied by several work-groups. Burman *et al.* investigated the effect of plant alcaloids on the structure and stability of hemoglobin using multispectroscopic, calorimetric, and molecular docking approaches, including CD spectroscopy [28]. Hasan *et al.* reported the efficient binding and inhibitory effect of gallic acid on fungal aspartic protease by disrupting its native structure as revealed by CD spectroscopic analysis [29]. Nedić *et al.* recommend far-UV CD and BeStSel analysis among the methods to study the structural effects of food antioxidants on plasma proteins [30].

Secondary structure composition was analyzed by BeStSel for globular proteins with different main components such as α-helical proteins or proteins rich in β-sheets [31–33]. In the field of intrinsically disordered proteins (IDPs), among other works, Sethi *et al.* showed that high-temperature incubation plays a key role in the micro- and nanoscale self-assembly of resilin-like disordered proteins; Nguyen *et al.* explored the structure of proteusin and identified disordered regions [34–37].

The capability of BeStSel to analyze the structure of protein aggregates and amyloid fibrils was used to investigate the inhibitory effects on the aggregation and toxic oligomer formation of Alzheimer’s amyloid-β peptide by vascular endothelial growth factor (VEGF) fragment [38], and by a snake venom protein-based inhibitor CDP-1 [39]. Park *et al.* worked out a strategic approach to designing flavonoids to target multiple pathological elements involved in AD by a complex methodology, including the effects on amyloid-β peptide secondary structure using CD and BeStSel [40]. Cytochrome *c* was also shown to redirect Zn(II)-bound amyloid-β peptide from amyloid fibril formation to amorphous aggregation under oxidative stress [41]. Inhibitory effect of cyclic-NDGA on γ-synuclein aggregation was studied by Singh *et al.* [42]. McCalpin *et al.* demonstrated that certain ganglioside lipids promote the aggregation of the islet amyloid polypeptide [43]. Aggregation of human insulin and the bacterial Hfq C-terminal region were also studied by CD combined with BeStSel [44, 45].

Nanotechnology and nanoparticle research is one of the most rapidly advancing scientific fields. BeStSel has been applied in several studies within this domain [46–48]. Peng *et al.* developed nanoparticle–drug complexes that enable controlled drug release after cancer surgery [49]. Domena *et al.* created carbon nanodots that enhance the accuracy of glioblastoma imaging [50].

Numerous works studied DNA- or RNA-binding proteins using CD with BeStSel. Yang *et al.* found that that four different small-molecule compound induced oligomers of amyotrophic lateral sclerosis (ALS) associated protein TDP-43 are stable, retain their DNA-binding ability, and have limited cytotoxicity. Despite their different morphology observed by TEM, CD spectroscopy revealed they have similar secondary structure composition [51]. Because these oligomers did not seed TDP-43 aggregation, the stabilization effect of the small-molecule compounds may offer a new approach to stop TDP-43 aggregation in various proteinopathies [51]. Ishiguro and Ishihama found that the ALS-associated protein TDP-43 plays a role in recognizing the RNA G-quadruplex, with mutations causing significant structural changes [52]. Kladova *et al.* investigated structural changes caused by mutations in SNP variants of the DNA polymerase β gene, revealing that certain mutations reduce or completely abolish enzymatic activity [53]. RNA-binding affinity of the RNA recognition domain (RRM) of histone lysine methyltransferase KMT2F was studied by Amin *et al.* CD spectroscopy revealed that the recombinant RRM of KMT2F exhibits a folded structure with significant α-helix content [54]. In engineering of invertebrate *Ramazzottius varieornatus* RvPolX DNA polymerase for biotechnological applications, structural analysis by CD spectroscopy aided to clarify the role of the N-terminal segment in the stability of the DNA–polymerase under high-salt conditions [55].

CD is frequently used to study protein–protein interactions. Border *et al.* investigated the interaction between Tau protein fibrils and TauRP(1–14) fragments [56]. Ye and Yu examined the effect of casein on various elemental fiber proteins, determining structural changes with BeStSel [57]. Similar studies have been conducted by Wei *et al.* [58] and by Pham *et al.* [59].

BeStSel is widely referenced not only for *in vitro* studies but also in *in silico* research, as bioinformatics methods have incorporated insights gained during its development [60, 61]. BeStSel has become an indispensable tool in CD spectroscopy. It is frequently cited in reviews and theoretical studies on CD [62–66].

### Case studies

**Figure 1.**
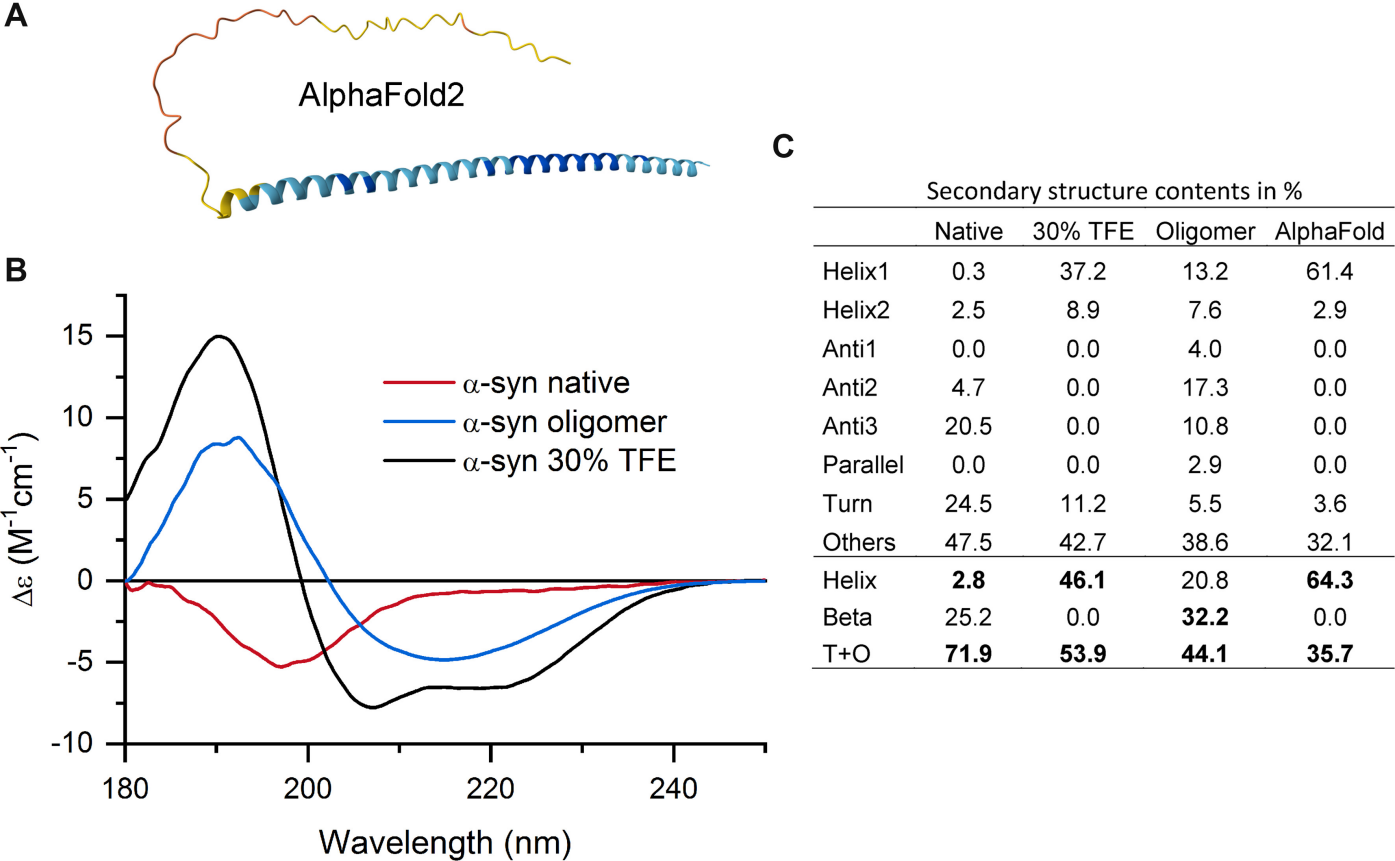
Investigation of the structure of α-synuclein by CD spectroscopy and validation of its AlphaFold prediction. (**A**) AlphaFold2 model of α-synuclein, predicting 64% α-helix structure with high confidence score (represented by blue color). (**B**) CD spectroscopy proves that the monomer α-synuclein under physiological conditions exhibits a CD spectrum characteristic of disordered structure. Moreover, α-synuclein migh form β-sheet rich oligomers, as well, and can take α-helical conformation in trifluoro-ethanol. These CD spectra were measured on a JASCO J-810 spectropolarimeter (JASCO Co., Tokyo, Japan) and used previously to present the *Disordered-Ordered Classification* tool of BeStSel [12]. (**C**) Secondary structure estimation by BeStSel for the α-synuclein forms in comparison to the secondary structure contents of the AlphaFold2 model.

CD spectroscopy with an accurate analysis tool provides a high throughput technique to verify if the protein is properly folded and exhibit the expected 3D structure under the environmental conditions used. The atomic-resolution structure to validate might be provided by an experimental technique, such as X-ray crystallography or NMR, or can be *in silico* model from MD simulations or AlphaFold [17]. AlphaFold is a revolutionary and high throughput technique to predict the protein structure; however, it does not account for experimental conditions, mutations, or post-translational modifications that can significantly impact protein structure, and thus requires experimental verification, preferably a rapid technique, such as CD combined with BeStSel. As illustrated here, the same polypeptide chain can adopt different conformations, which AlphaFold cannot predict. α-Synuclein is a natively disordered protein linked to Parkinson’s disease and other synucleinopathies. AlphaFold erroneously predicts a high α-helix content with high confidence (Fig. 1A), whereas CD confirms that the protein is actually disordered under native conditions (Fig. 1B). It can also form β-sheet-rich oligomers and, under extreme conditions with high triflouroethanol concentrations, it adopts an α-helical structure, though to a lesser extent than AlphaFold predicted (Fig. 1C). Upon binding to lipid membranes, α-synuclein molecule was reported to adopt α-helical structures [67–69].

**Figure 2.**
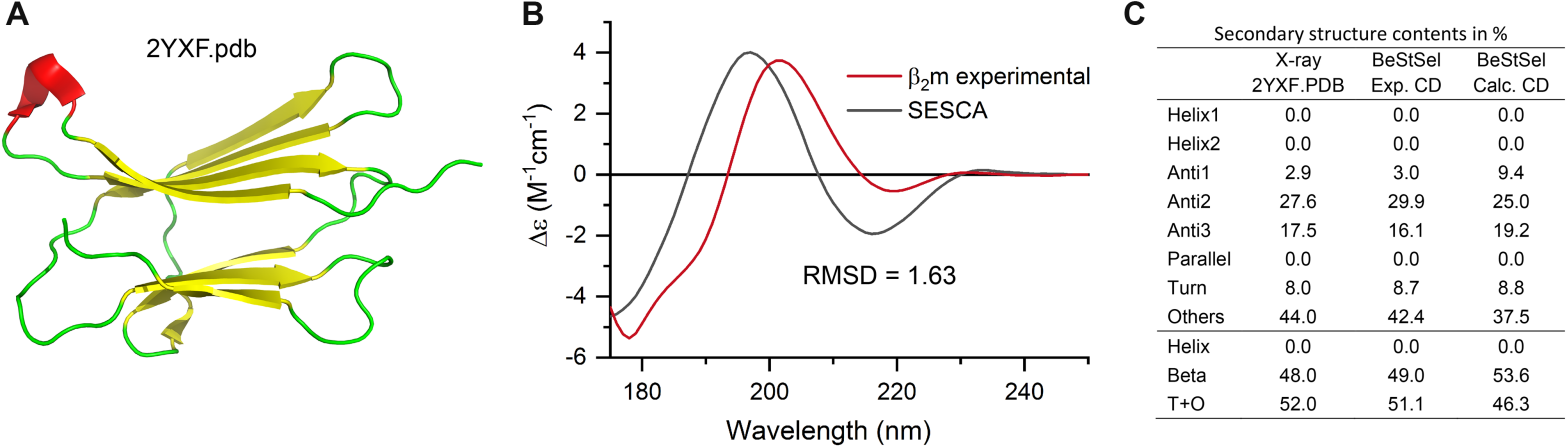
Validation of the structure of recombinant β_2_-microglobulin (β2m) by CD spectroscopy. (**A**) X-ray structure (2YXF) of β2m consisting of mainly antiparallel β-sheets [72]. (**B**) Experimental CD spectrum of the protein recorded by SRCD at the DISCO beamline, SOLEIL Synchrotron [3, 11], and CD spectrum calculated *in silico* for the 2YXF.pdb structure by the SESCA algorithm [70]. Two validation methods are presented here. As the goal is to validate the structure of the recombinant protein, the experimental CD spectrum was analyzed by BeStSel and compared to the secondary structure derived from the X-ray structure by using the “*Secondary structure from PDB files*” function of the BeStSel web server, showing good agreement (**C**). Another method is to match the experimental CD to a calculated CD spectrum [70, 71]. We observe significant spectral differences and large RMSD between them (B), which we might interpret so that the structures behind are different, i.e. the measured protein structure is different from the one deposited in the PDB. For curiosity, we analyzed the calculated CD spectrum with BeStSel, and it showed a similar secondary structure, although with some differences, to the X-ray structure and to the results of the experimental data analysis (C).

There are different potential approaches suggested to validate the high-resolution structures or structural models. One, direct approach is to measure the CD spectrum of the protein, analyze it for the secondary structure composition (e.g. using BeStSel) and compare the results to the 3D-structure. For the comparison, we need to know the secondary structure contents of the 3D-structure (calculation provided on the BeStSel web server). The other approach is the *in silico* calculation of CD spectra for the structural models and comparison to the experimentally measured CD spectrum to validate or select the suitable structural model, based on the RMSD between the spectra. Calculation of CD spectrum from the 3D-structure is challenging task, SESCA [70] and PDB2CD [71] algorithms might be used. The spectral differences are difficult to interpret, behind a similar spectral RMSD there can be deviations in different regions of the spectra contributed by different structural components; moreover, the different secondary structure components contribute to the spectrum with different amplitudes. Users of these spectrum-calculating tools often end-up analyzing their spectra with BeStSel or other tools for secondary structure contents for a better comparison. Instead of spectral comparison, we recommend the direct comparison of the secondary structures estimated for the experimental CD spectrum to the model structures. Figure 2 compares the two approaches on the example of β_2_-microglobulin [72], the light chain of MHC-I, which is associated with dialysis related amyloidosis in long term dialysis patients [73].

## New features

Basis matrices: Instead of fixed basis spectra sets, we introduced a new system with “basis matrices”, which handle the spectral contributions of the different secondary structure elements depending on the secondary structure composition of the protein (see the “Materials and methods” section). The new algorithm improved the secondary structure estimation accuracy (Table 1 and Supplementary Table S1).

Smoothing and NRMSD: The updated BeStSel version applies smoothing with a 2 nm window before secondary structure analysis. While this has minimal impact on smooth, noiseless spectra, it significantly enhances performance for noisy spectra, which often occur at lower wavelengths. Users applying their own smoothing are advised to avoid larger windows, as excessive smoothing may distort the spectrum and compromise the accuracy of structure estimation. To better assess fitting reliability, BeStSel introduces an improved NRMSD parameter. In noisy spectra, RMSD and NRMSD values between the experimental and fitted spectra tend to be inflated due to noise, which can obscure the true performance of the method. To mitigate this, NRMSD is calculated using the smoothed spectrum compared to the fitted spectrum, reflecting better the reliability of BeStSel fitting. RMSD, however, remains calculated from the original spectrum, preserving sensitivity to noise levels. Supplementary Figs S1-S4 give an explanation for the new NRMSD and compare it to the previous one.

The *Fold prediction* module was upgraded from CATH 4.3 to CATH 4.4 data [4], which increased the number of domains from 500 238 to 601328. The number of Architectures and Topologies are increased from 41 and 1390 to 43 and 1472, respectively.

The PDB database used for the *Fold recognition* and to *Secondary structure from PDB files* by the four letter PDB codes was refreshed by adding 38 493 new structures representing 259 021 new polypeptide chains. Thus, the present number of PDB structures and chains are 213 856 and 762 493, respectively.

The “*Secondary structure from PDB files”* module was updated, calculation for mmCIF and AlphaFold structures is available for comparison to the results of BeStSel analysis of experimental CD spectra.

*Thermal denaturation analysis* is a new module of the web server. Thermal denaturation of proteins can be followed by recording the CD signal at a constant wavelength as a function of temperature. Such data can be uploaded to the web server and fitted by a two-state model based on the Gibbs–Helmholtz equation. The analysis provides the melting temperature (*T*_m_), the denaturation enthalpy change at the melting temperature (Δ*H*_m_) and other parameters of the melting curve, such as the slopes before and after the transition and the CD values for the native and denature state, extrapolated to 0°C. Knowing the Δ*C*_p_ (measured or estimated) value, the Δ*G°* thermodynamic stability of the protein can be calculated using the determined *T*_m_ and Δ*H*_m_ values.

The tutorial that can be found under the “*Documentation*” tab has been updated and can be downloaded. Information and help is provided upon using the web server.

## Limitations and further developments

The eight secondary structure components of BeStSel do not account for polyproline-II helices (typical of collagen-like structures), specific turn types (prevalent in short peptides), or 3_10_-helices, which occur in some globular proteins, limiting its applicability to such structures. BeStSel also excludes aromatic contributions (as do other algorithms), which may impact results when aromatic residues are abundant.

Although the new algorithm provides improved accuracy for disordered structures, in highly disordered proteins, part of the structure may be misclassified as highly right-twisted antiparallel β-sheet (Anti3) due to spectral similarities [3, 9].

BeStSel surpasses earlier methods by enabling the structural estimation of β-sheet-rich protein aggregates and amyloid fibrils [3]. However, accurate analysis may be hindered by spectral artifacts arising from differential light scattering, precipitation, or linear dichroism in such samples. For details how to investigate such samples by CD spectroscopy, we recommend our protocol paper in *Tools2025* special issue of *Protein Science* [74].

Our eternal future goal is to increase the reference database and further improve structure prediction, especially for disordered proteins.

## Conclusions

The BeStSel web server provides an advanced tool for analyzing protein CD spectra, offering accurate secondary structure determination and protein fold prediction. By resolving eight secondary structure components, including detailed β-structure features (orientation and twist), BeStSel achieves superior accuracy compared to previous methods and is particularly effective for β-sheet-rich proteins, protein aggregates, and membrane proteins. Users can analyze single or multiple spectra, adjust wavelength ranges, scale spectra, and access graphical and text-based output for streamlined analysis. Secondary structure calculations for 3D structures facilitate the validation of MD and AlphaFold models, while a dedicated module distinguishes IDPs from ordered ones. Additionally, the newly implemented thermal denaturation analysis allows the extraction of melting points and thermodynamic parameters from CD thermal scans. Supporting high-throughput analysis, the server provides a robust and versatile platform for protein CD spectroscopy, serving diverse applications in structural biology, biotechnology, and the pharmaceutical sciences.

## Supplementary Material

gkaf378_Supplemental_File

## Data Availability

The BeStSel web server is freely accessible at https://bestsel.elte.hu. The datasets generated for this study are available on request to the corresponding author.
