# Supplementary material for: BeStSel: analysis site for protein CD spectra—2025 update
Source: Nucleic Acids Res. 2025 May 13;53(W1):W73–83. doi: 10.1093/nar/gkaf378 (PMC12230724; doi:10.1093/nar/gkaf378)
Supplement: gkaf378_Supplemental_File [file gkaf378_supplemental_file.pdf]

Supplementary Information

BeStSel: analysis site for protein CD spectra – 2025 Update

András Micsonai, Frank Wien, Nikoletta Murvai, Péter Márton Nyiri, Bori Balatoni, Young-Ho Lee, Tamás Molnár, Yuji Goto, Frédéric Jamme, and József Kardos

Contents:

Page 2: Table S1. Comparison of the performance of different methods for secondary structure estimation. Test on an independent set of β-sheet-rich or rare structures

Page 3: Explanation for the effect of noise and wavelength range on BeStSel performance (examples) and the new NRMSD

Page 4: Figure S1. Effect of spectral noise on BeStSel analysis of an α-helical protein in the 175-250 nm wavelength range.

Page 5: Figure S2. Effect of spectral noise on BeStSel analysis of an α-helical protein in the 195-250 nm wavelength range.

Page 6: Figure S3. Effect of spectral noise on BeStSel analysis of a protein with β-structure in the 175-250 nm wavelength range.

Page 7: Figure S4. Effect of spectral noise on BeStSel analysis of a protein with β-structure in the 195-250 nm wavelength range.

Page 8: References for SI

Table S1. Comparison of the performance of different methods for secondary structure estimation. Test on an independent set of β-sheet-rich or rare structures.[a]

		Helix		Antiparallel		Parallel		β-sheet		Turn+Others	
Method	Failures[b]	RMSD[c]	Corr[d]	RMSD	Corr	RMSD	Corr	RMSD	Corr	RMSD	Corr
BeStSel 2025	-	0.035	0.99	0.046	0.97	0.031	0.97	0.034	0.99	0.038	0.91
BeStSel 2022	-	0.034	0.99	0.049	0.97	0.037	0.97	0.035	0.99	0.038	0.91
VARSLC	5	0.089	0.97	0.155	0.62	0.860	-0.08	0.133	0.73	0.130	0.74
LINCOMB	-	0.119	0.91	0.214	0.45	0.198	0.59	0.230	0.51	0.232	0.59
CDNN	-	0.083	0.97	0.122	0.83	0.076	0.91	0.102	0.89	0.115	0.81
SELCON	-	0.147	0.86					0.122	0.82	0.077	0.73
CONTIN	2	0.095	0.95					0.068	0.96	0.074	0.73
CDSSTR	-	0.201	0.76					0.139	0.75	0.099	0.71
K2D	-	0.198	0.84					0.152	0.79	0.153	0.55
K2D2	-	0.222	0.70					0.162	0.71	0.088	0.68
K2D3	-	0.136	0.87					0.184	0.64	0.143	0.65
CAPITO	-	0.260	0.57					0.161	0.85	0.147	0.70

[a]Performance of different algorithms on a set of CD spectra of 25 proteins, independent of SP175+ reference dataset, that are either rich in β-sheets or have high α-helical content, or rare structural composition (1). The performance of the previous version of the BeStSel algorithm is from Micsnai et al. (2) and other algorithms are from Micsonai et al. (1). The results for SELCON, CONTIN, CDSSTR (3), LINCOMB (4) are cross-validated, but for CDNN (5), CAPITO (6), VARSLC (7), and K2Ds(8,9)) are not cross-validated. [b]For some spectra, some algorithms could not complete the calculation and froze or gave error messages. [c]Root-mean-square-deviation, [d]Pearson-correlation coefficient.

Effect of noise and wavelength range on BeStSel performance (examples) and the new NRMSD

The updated BeStSel version applies smoothing with a 2 nm window before secondary structure analysis. While this has minimal impact on smooth, noiseless spectra, it significantly enhances performance for noisy spectra, which often occur at lower wavelengths. Here, we present a study on the effect of spectral noise, and the effect of smoothing on the secondary structure estimation of BeStSel. We introduced a new NRMSD[1] calculated between the smoothed spectrum and the fitted spectrum, reflecting better the reliability of BeStSel fitting.

Here, we investigate the CD spectra of two proteins, one α-helical and another β-structured, both in two wavelength ranges, 175-250 nm and 195-250 nm. The BeStSel analyses are shown for the original noiseless spectra and for the same protein with additional noise at three levels, 0.5, 1, and 2 $M^{-1}cm^{-1}$. The added noise is random noise with normal distribution, zero mean, with the given deviation. CD data with 0.1 nm data steps are used as initial input spectra. Supplementary Figure S1, S2 show the spectra and BeStSel analyses of the α-helical protein in the wavelength range of 175-250 nm and 195-250 nm, respectively. Supplementary Figure S3, S4 present the CD spectra and BeStSel analyses of the β-structured protein in the wavelength range of 175-250 nm and 195-250 nm, respectively. In all figures, panels A-D show the spectra with different noise, the smoothed spectra and the fitted spectra. For clarity and better comparison, panels E show the fitted results from A-D. We have to notice that the smoothed spectra in panels A-D are close to each other and the fitted spectra are similar, despite the increasing noise of the input spectra. The resulting secondary structure estimations shown at panel G are also quite similar, with somewhat decreasing accuracy with the increasing noise. To show better the reliability of BeStSSel fitting, the NRMSD was calculated between the smoothed and the fitted spectra. This way the large effect of the noise on the NRMSD was avoided. Panel F shows this NRMSD (new NRMSD) compared to the previous NRMSD calculated between the input spectra extracted with 1 nm data step and the fitted spectra (for the same BeStSel analyses using initial smoothing). Notably, the previous and the new NRMSD values are close to each other for smooth, noiseless spectra and the new RMSD exhibits only minor increase with noise when the fit is still accurate. Panels G and H compare the estimated secondary structure contents to the X-ray structure of the proteins applying BeStSel with and without the initial smoothing, respectively. The performance of BeStSel proved to be relatively good for the broad wavelength range of 175-250 nm (Fig.S1 and S3), especially for the α-helical protein. However, in the range of 195-250 nm (Fig. S2 and S4), without the smoothing step, there is a significant decrease in accuracy with increasing noise. The results for the β-structured protein are unacceptable for the noisy spectra without initial smoothing (Fig. S4H).

[1] NRMSD of spectral fit in general is calculated as follows:

$$NRMSD = sqrt\left(\frac{\sum_{i=1}^{w}\left(CD_{exp,i}-CD_{fit,i}\right)^2}{w\cdot\left(\max(CD_{exp})-\min(CD_{exp})\right)^2}\right)$$

where w is the number of data points.

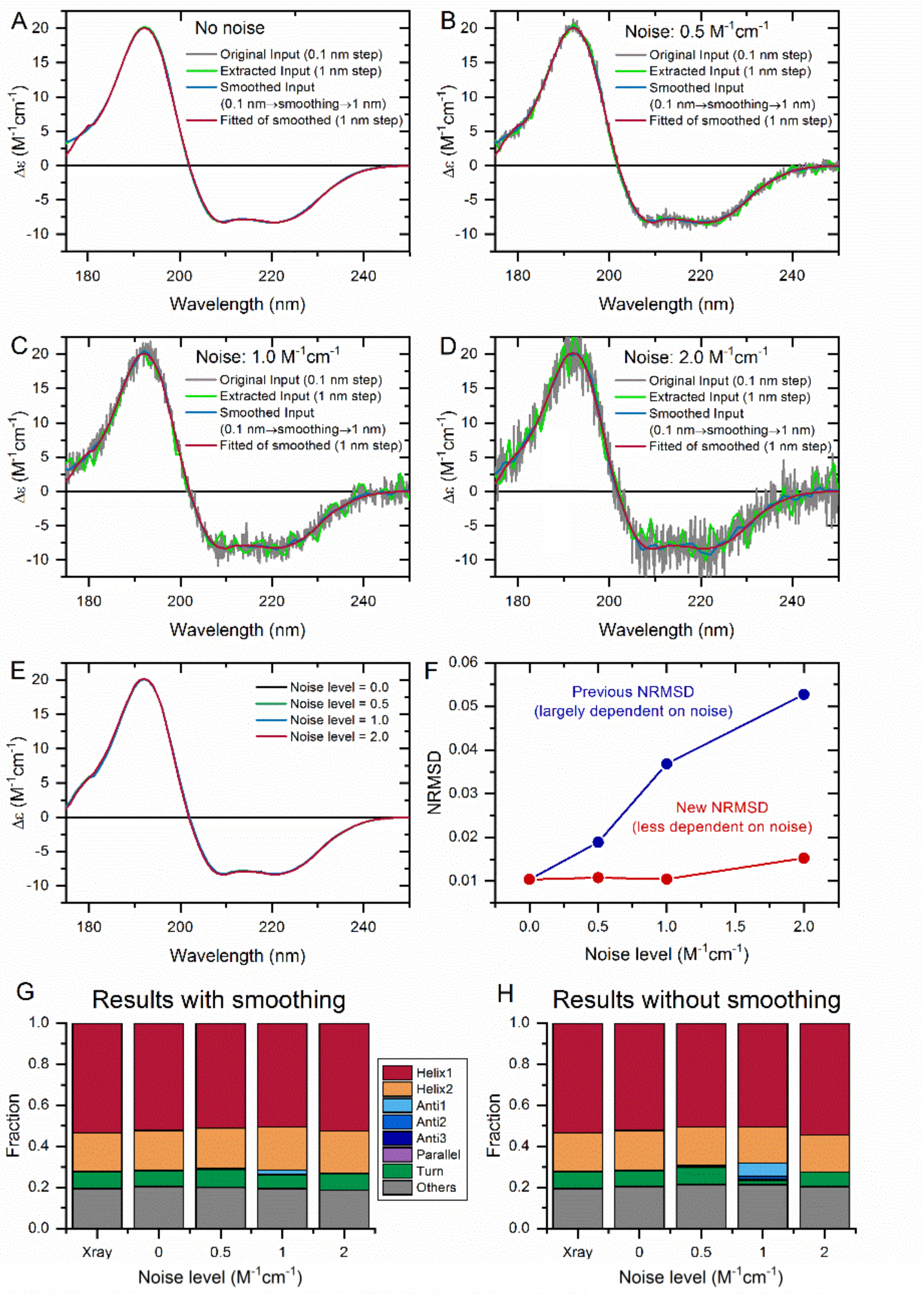

Figure S1. Effect of spectral noise on BeStSel analysis of an α-helical protein in the 175-250 nm wavelength range. (A-D) Spectra with no noise and with 0.5, 1.0, and 2.0 $M^{-1}cm^{-1}$ noise level, respectively. Smoothed and fitted spectra are also shown. (E) Fitted spectra from panels A-D, (F) NRMSD values calculated between the noisy input spectra and the fitted ones (previous method) and between the smoothed and the fitted spectra (new NRMSD). (G) BeStSel secondary structure estimation results with initial smoothing and (H) without smoothing.

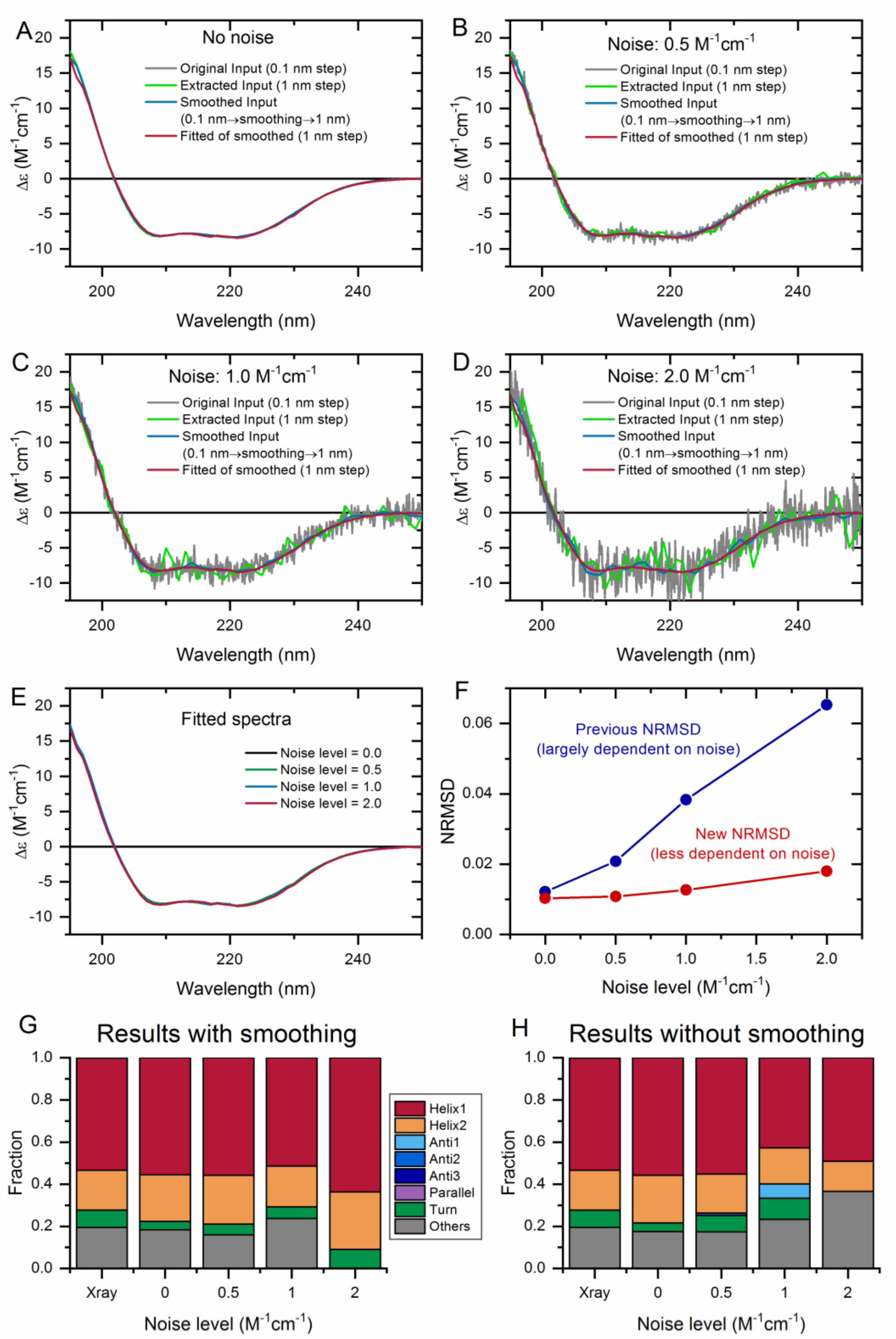

Figure S2. Effect of spectral noise on BeStSel analysis of an α-helical protein in the 195-250 nm wavelength range. (A-D) Spectra with no noise and with 0.5, 1.0, and 2.0 $M^{-1}cm^{-1}$ noise level, respectively. Smoothed and fitted spectra are also shown. (E) Fitted spectra from panels A-D, (F) NRMSD values calculated between the noisy input spectra and the fitted ones (previous method) and between the smoothed and the fitted spectra (new NRMSD). (G) BeStSel secondary structure estimation results with initial smoothing and (H) without smoothing.

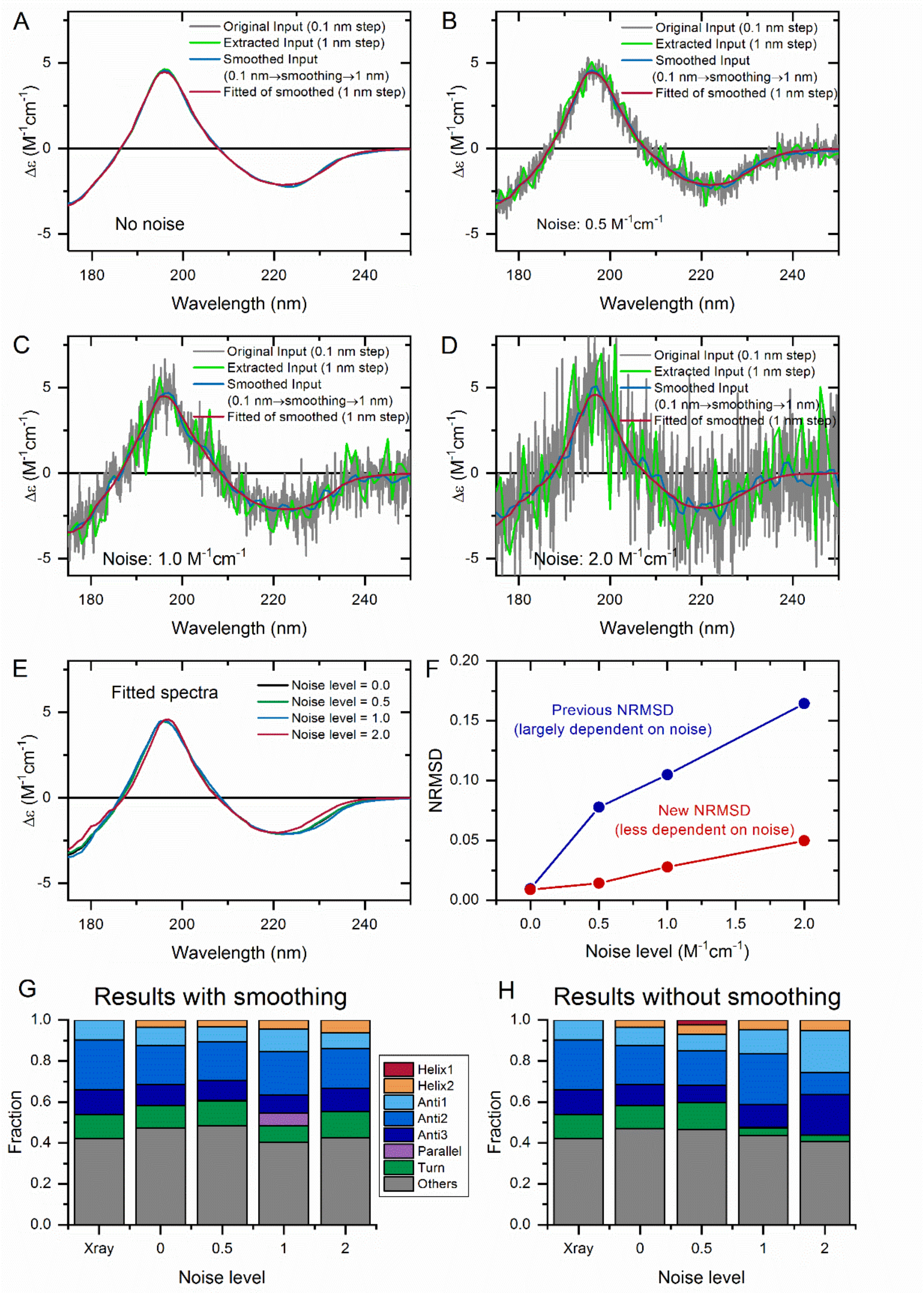

Figure S3. Effect of spectral noise on BeStSel analysis of a protein with β-structure in the 175-250 nm wavelength range. (A-D) Spectra with no noise and with 0.5, 1.0, and 2.0 $M^{-1}cm^{-1}$ noise level, respectively. Smoothed and fitted spectra are also shown. (E) Fitted spectra from panels A-D, (F) NRMSD values calculated between the noisy input spectra and the fitted ones (previous method) and between the smoothed and the fitted spectra (new NRMSD). (G) BeStSel secondary structure estimation results with initial smoothing and (H) without smoothing.

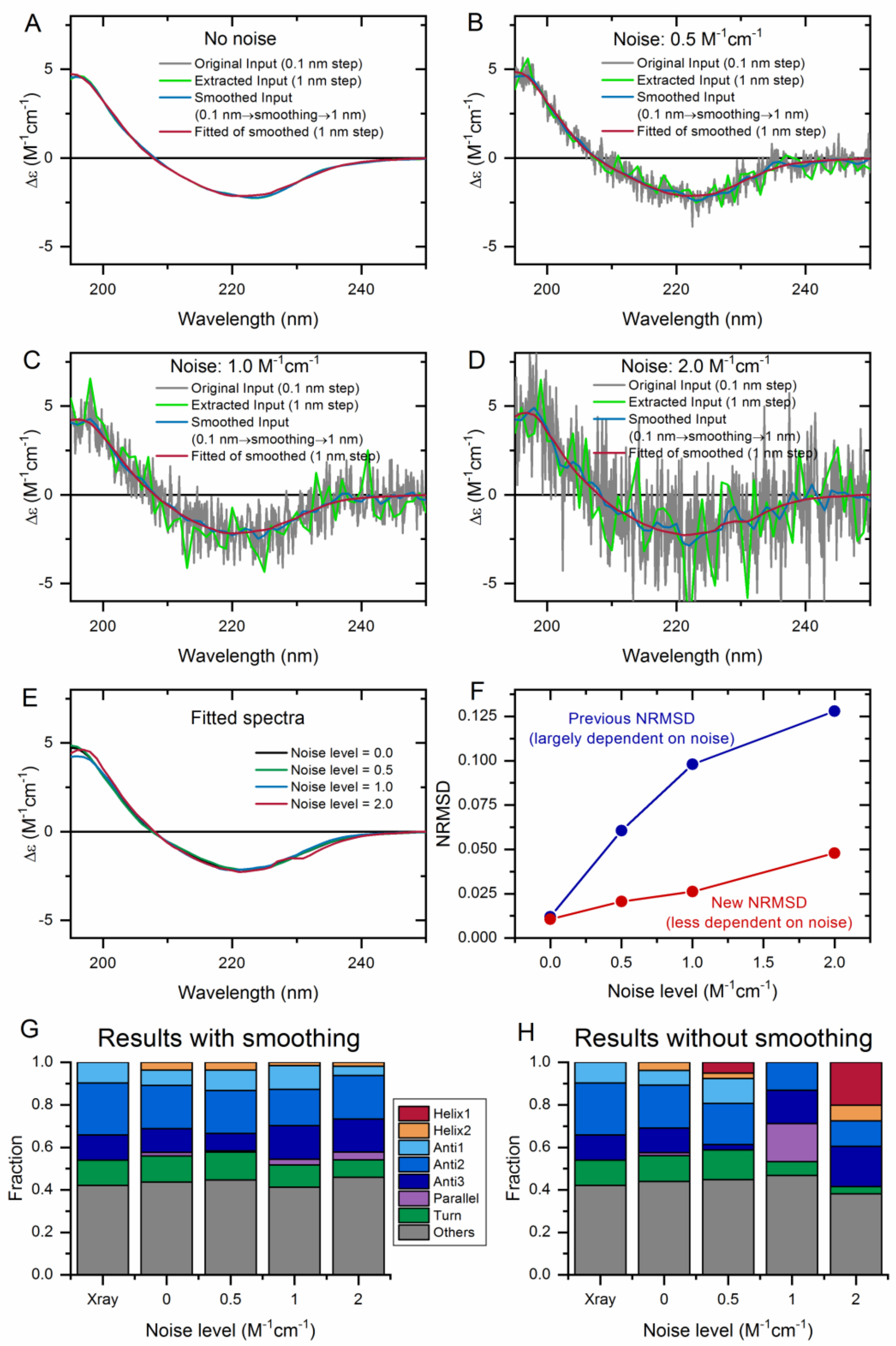

Figure S4. Effect of spectral noise on BeStSel analysis of a protein with β-structure in the 195-250 nm wavelength range. (A-D) Spectra with no noise and with 0.5, 1.0, and 2.0 $M^{-1}cm^{-1}$ noise level, respectively. Smoothed and fitted spectra are also shown. (E) Fitted spectra from panels A-D, (F) NRMSD values calculated between the noisy input spectra and the fitted ones (previous method) and between the smoothed and the fitted spectra (new NRMSD). (G) BeStSel secondary structure estimation results with initial smoothing and (H) without smoothing. Note, that without initial smoothing, the results are unacceptable at higher noise levels.

1. Micsonai, A., Wien, F., Kernya, L., Lee, Y.H., Goto, Y., Refregiers, M. and Kardos, J. (2015) Accurate secondary structure prediction and fold recognition for circular dichroism spectroscopy. *Proc Natl Acad Sci U S A*, **112**, E3095-3103.

http://www.ncbi.nlm.nih.gov/pubmed/26038575

http://dx.doi.org/10.1073/pnas.1500851112

2. Micsonai, A., Moussong, E., Wien, F., Boros, E., Vadaszi, H., Murvai, N., Lee, Y.H., Molnar, T., Refregiers, M., Goto, Y. *et al.* (2022) BeStSel: webserver for secondary structure and fold prediction for protein CD spectroscopy. *Nucleic Acids Res*, **50**, W90-W98.

http://www.ncbi.nlm.nih.gov/pubmed/35544232

http://dx.doi.org/10.1093/nar/gkac345

3. Sreerama, N. and Woody, R.W. (2000) Estimation of protein secondary structure from circular dichroism spectra: comparison of CONTIN, SELCON, and CDSSTR methods with an expanded reference set. *Anal Biochem*, **287**, 252-260.

http://www.ncbi.nlm.nih.gov/pubmed/11112271

4. Toumadje, A., Alcorn, S.W. and Johnson, W.C., Jr. (1992) Extending CD spectra of proteins to 168 nm improves the analysis for secondary structures. *Anal Biochem*, **200**, 321-331.

http://www.ncbi.nlm.nih.gov/pubmed/1632496

5. Provencher, S.W. and Glockner, J. (1981) Estimation of globular protein secondary structure from circular dichroism. *Biochemistry*, **20**, 33-37.

http://www.ncbi.nlm.nih.gov/pubmed/7470476

6. Wiedemann, C., Bellstedt, P. and Gorlach, M. (2013) CAPITO--a web server-based analysis and plotting tool for circular dichroism data. *Bioinformatics*, **29**, 1750-1757.

http://www.ncbi.nlm.nih.gov/pubmed/23681122

7. Manavalan, P. and Johnson, W.C., Jr. (1987) Variable selection method improves the prediction of protein secondary structure from circular dichroism spectra. *Anal Biochem*, **167**, 76-85.

http://www.ncbi.nlm.nih.gov/pubmed/3434802

8. Perez-Iratxeta, C. and Andrade-Navarro, M.A. (2008) K2D2: estimation of protein secondary structure from circular dichroism spectra. *BMC Struct Biol*, **8**, 25.

http://www.ncbi.nlm.nih.gov/pubmed/18477405

9. Louis-Jeune, C., Andrade-Navarro, M.A. and Perez-Iratxeta, C. (2011) Prediction of protein secondary structure from circular dichroism using theoretically derived spectra. *Proteins*, **80**, 374-381.

http://www.ncbi.nlm.nih.gov/pubmed/22095872
